# High electron mobility and low carrier concentration of hydrothermally grown ZnO thin films on seeded *a*-plane sapphire at low temperature

**DOI:** 10.1186/s11671-014-0715-0

**Published:** 2015-01-22

**Authors:** Nurul Azzyaty Jayah, Hafizal Yahaya, Mohamad Rusop Mahmood, Tomoaki Terasako, Kanji Yasui, Abdul Manaf Hashim

**Affiliations:** Malaysia-Japan International Institute of Technology, Universiti Teknologi Malaysia, Jalan Sultan Yahya Petra, Kuala Lumpur, 54100 Malaysia; Faculty of Electrical Engineering, Universiti Teknologi MARA, Shah Alam, Selangor 40450 Malaysia; Graduate School of Science and Engineering, Ehime University, Ehime, 790-8577 Japan; Department of Electrical Engineering, Nagaoka University of Technology, Kamitomioka-machi, Nagaoka, Niigata 940-2188 Japan

**Keywords:** Hydrothermal deposition, Seed layer, Mobility, Carrier concentration, Zinc oxide

## Abstract

Hydrothermal zinc oxide (ZnO) thick films were successfully grown on the chemical vapor deposition (CVD)-grown thick ZnO seed layers on *a*-plane sapphire substrates using the aqueous solution of zinc nitrate dehydrate (Zn(NO_3_)_2_). The use of the CVD ZnO seed layers with the flat surfaces seems to be a key technique for obtaining thick films instead of vertically aligned nanostructures as reported in many literatures. All the hydrothermal ZnO layers showed the large grains with hexagonal end facets and were highly oriented towards the *c*-axis direction. Photoluminescence (PL) spectra of the hydrothermal layers were composed of the ultraviolet (UV) emission (370 to 380 nm) and the visible emission (481 to 491 nm), and the intensity ratio of the former emission (*I*_UV_) to the latter emission (*I*_VIS_) changed, depending on both the molarity of the solution and temperature. It is surprising that all the Hall mobilities for the hydrothermal ZnO layers were significantly larger than those for their corresponding CVD seed films. It was also found that, for the hydrothermal films grown at 70°C to 90°C, the molarity dependences of *I*_UV_/*I*_VIS_ resembled those of mobilities, implying that the mobility in the film is affected by the structural defects. The highest mobility of 166 cm^2^/Vs was achieved on the hydrothermal film with the carrier concentration of 1.65 × 10^17^ cm^−3^ grown from the aqueous solution of 40 mM at 70°C.

**Electronic supplementary material:**

The online version of this article (doi:10.1186/s11671-014-0715-0) contains supplementary material, which is available to authorized users.

## Background

Since a few decades ago, zinc oxide (ZnO), a transparent conducting metal oxide, has been intensively and widely studied for the fabrication of many kinds of devices due to its unique properties such as wide direct bandgap energy (3.37 eV), large exciton binding energy (60 meV), and thermal stability [1-3]. Beside a traditional film-layered structure, various morphologies of ZnO nanostructures, i.e., nanowires (NWs), nanorods (NRs), nanoflowers (NFs), and nanotubes (NTs) [1,4,5], offer unique possibilities for use in many applications, mainly in the field of electronics, optics, and photonics [2-4,6-11]. However, due to the lack of a low-cost lattice-matched epitaxy substrate, the growth of ZnO films and nanostructures is commonly done on a *c*-plane sapphire and silicon carbide (SiC) substrate [12,13]. Many efforts have been made previously to achieve good ZnO structures, and it seems to show that the effects of the seed/buffer layer either in the form of homo- or hetero-structure and the deposition method are very prominent. For example, graphene and magnesium oxide (MgO) have been used as the hetero-seed/buffer layers to grow ZnO structures on an insulator and a sapphire substrate, respectively [14,15]. Meanwhile, the properties of a homo-seed/buffer layer, i.e., ZnO seed, have also been shown to give significant differences on the morphologies and properties of the subsequent deposited ZnO structures [11,16,17]. Of course, the method of subsequent deposition of ZnO itself will also give significant differences on the morphologies and properties of the grown structures.

Vapor-phase deposition techniques are widely used to grow both the seed/buffer layers and ZnO films with high electron mobility and low carrier concentration. Kaidashev et al. presented a multistep pulsed-laser deposition to achieve electron mobility up to 155 cm^2^/Vs in a narrow carrier concentration range from 2 to 5 × 10^16^ cm^−3^ with insertion of a 30-nm-thin ZnO buffer layer on a *c*-plane sapphire substrate [13]. Tampo et al. reported an enhancement of electron mobility in a ZnMgO/ZnO/MgO/*c*-plane sapphire hetero-structure grown by radical source molecular beam epitaxy (MBE) due to the formation of a two-dimensional electron gas (2DEG) [15]. As a result, a high electron mobility of 250 cm^2^/Vs at room temperature (RT) and a low sheet carrier concentration of 1 × 10^13^ cm^−2^ were achieved. Chu et al. presented the growth of a ZnO thin film on *c*-plane sapphire with a relatively high electron mobility of 169 cm^2^/Vs and a low carrier concentration of 2 × 10^16^ cm^−3^ using plasma-assisted MBE by optimizing the thickness of a MgO buffer layer [12]. To our knowledge, the highest mobility of 137 cm^2^/Vs together with a minimum carrier concentration of 7 × 10^16^ cm^−3^ was obtained for a ZnO thin film grown directly on *a*-plane sapphire without the introduction of any seed layer by using MBE as reported by Fons et al. [18]. However, these vapor-phase techniques have some drawbacks such as expensive apparatus, complicated procedure, low yield, and high temperature.

Liquid-phase techniques such as hydrothermal and electrochemical processes have been widely used to grow ZnO structures on ZnO-seeded substrates [7,11,17,19-21], catalyzed substrates [22], or seed/catalyst-free substrates [14,19,21,23]. Generally, the grown ZnO structures on the ZnO-seeded substrates are mostly in the form of one-dimensional (1D) nanostructures [7,9,20,21,24,25] due to the nature of chemical reaction of liquid-phase techniques and also due to the small grains and rough surfaces of ZnO seed layers prepared by the sputtering method [7,20,21] or other deposition techniques [9,24,25]. Song et al. presented the effects of a seed layer prepared by the sputtering method on the growth of ZnO NRs [17]. Nanosized metal catalyzers have also been proven to be able to assist the growth of ZnO 1D nanostructures. Li et al. reported on the synthesis of ZnO nanoparticles on Si substrates with and without an Au catalyst [16]. Recently, we report the growth of ZnO nanostructures on seed/catalyst-free substrates by introducing graphene as the template layer using both electrochemical and hydrothermal processes [14,23,26]. Up to date, there is no report on the formation of a ZnO continuous film structure on a ZnO-seeded substrate using liquid-phase methods. In this paper, we report the growth of ZnO continuous film structure on chemical vapor deposition (CVD)-grown ZnO seeds on *a*-plane sapphires by a hydrothermal process. In this study, higher mobilities and lower carrier concentrations of the hydrothermally grown ZnO layer as compared to the original values of ZnO seeds were observed. These results suggest that the ZnO layer with low carrier concentration can be realized by a hydrothermal deposition, resulting to the increase of carrier mobility.

## Methods

A thick CVD-grown ZnO layer on *a*-plane sapphire with thicknesses ranging from 2.0 to 5.0 μm was used as the substrate as reported by Yasui et al. [27]. The substrate was placed facing downwards in the middle of a Teflon-made holder inside a glass beaker containing an equimolar solution of zinc nitrate dehydrate Zn(NO_3_)_2_ and hexamethylenetetramine (HMTA) (CH_2_)_6_N_4_ which were dissolved in 100 ml of deionized (DI) water. The molarities of solutions were set to 2, 20, 30, and 40 mM, while the growth temperatures were set to 70°C, 80°C, and 90°C. The growth was carried out for 3 h after the temperature of the aqueous solution reached the set temperatures, i.e., 70°C, 80°C, and 90°C. Finally, the sample was taken out from the aqueous solution and immersed into DI water to remove any residue on the substrate. The samples were characterized using atomic force microscopy (AFM, XE-100 Park Systems), field emission scanning electron microscopy (FESEM, Hitachi SU8030), X-ray diffraction (XRD, Bruker AXES D8 Advance), photoluminescence (PL, manually setup system) spectroscopy [28], and Hall effect measurement (Ecopia-5300).

## Results and discussion

**Figure 1:**
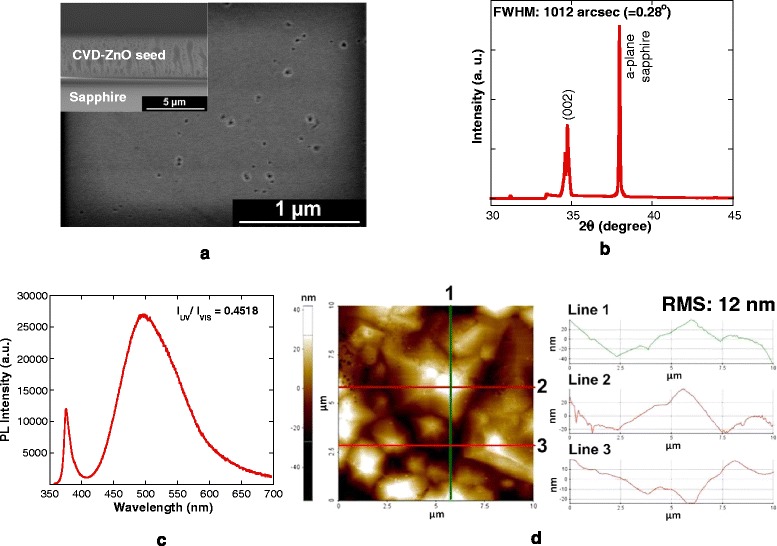
**FESEM and AFM images and XRD and RT PL spectra of the CVD ZnO seed. (a)** FESEM images of top and cross-sectional views, **(b)** XRD 2*θ* scan spectra, **(c)** RT PL measurement of the CVD ZnO seed sample, and **(d)** AFM image with line profiles of the CVD ZnO seed.

Figure 1a shows an example of an FESEM image of the CVD ZnO seed used in the hydrothermal growth. The morphology shows a continuous film structure with a relatively smooth surface. The ZnO seeds used in this work are thick, and their thicknesses are in the range of 2.0 to 5.0 μm. Relatively thick CVD ZnO seeds were purposely chosen in order to minimize the effects of seed thickness on the hydrothermally grown ZnO structures. As reported by Song et al. [17], a significant change in the ZnO NR growth was not found for sputtered ZnO seeds with thicknesses between 330 and 950 nm. They also reported that the decrease in the diameter of the ZnO NRs with decreasing seed layer thickness is due to the smaller crystal or grain size of the ZnO seed layer. Therefore, we speculate that the effect of seed thickness can be ignored by using a thick seed layer. The surface of the thick ZnO seed should also be sufficiently relaxed from any severe lattice stress contributed from the large lattice mismatch between sapphire and ZnO. Consequently, this allows the possible effect that is related to the fluctuation in the seed thicknesses among the samples to be ignorable, and the comparison of the hydrothermally grown layers at different temperatures and molarities can be done in a more precise manner. Thus, the properties as well as the potential merit of the hydrothermally deposited layer can be precisely revealed and determined. Figure 1b shows an example of the XRD spectrum of the CVD ZnO seed. The observation of a strong (002) peak indicates that the ZnO seed has a hexagonal wurtzite structure and is highly oriented along the *c*-axis. Figure 1c shows an example of the room temperature (RT) PL spectrum of the CVD ZnO seed, where two distinct peaks were observed. The first peak is located in the ultraviolet (UV) region (370 to 380 nm) which corresponds to near-band-edge (NBE) emission while the second peak appears in a blue-green emission region (480 to 500 nm) of the visible spectrum. The relative RT PL intensity ratio of the emission in the UV region to the emission in the visible region, *I*_UV_/*I*_VIS_, is confirmed to be in the range of 0.35 to 0.65. The respective AFM topographical image of the CVD ZnO seed together with the measured line profiles is shown in Figure 1d. It can be seen that the top surface of the CVD ZnO seed shows large grain structures where the diameter and height of grain structures were confirmed to be more than 5 μm and below 100 nm, respectively. Based on the ratio of the height and the radius of grain structures, it can be said that the slopes of the structures are relatively very low and are close to a flat condition. Such a structure is significantly different with the sputtered ZnO seed, and such characteristic is believed to promote the lateral growth of ZnO structures by the subsequent hydrothermal deposition, resulting to the formation of a film structure. The growth mechanism is discussed in the next section.

**Figure 2:**
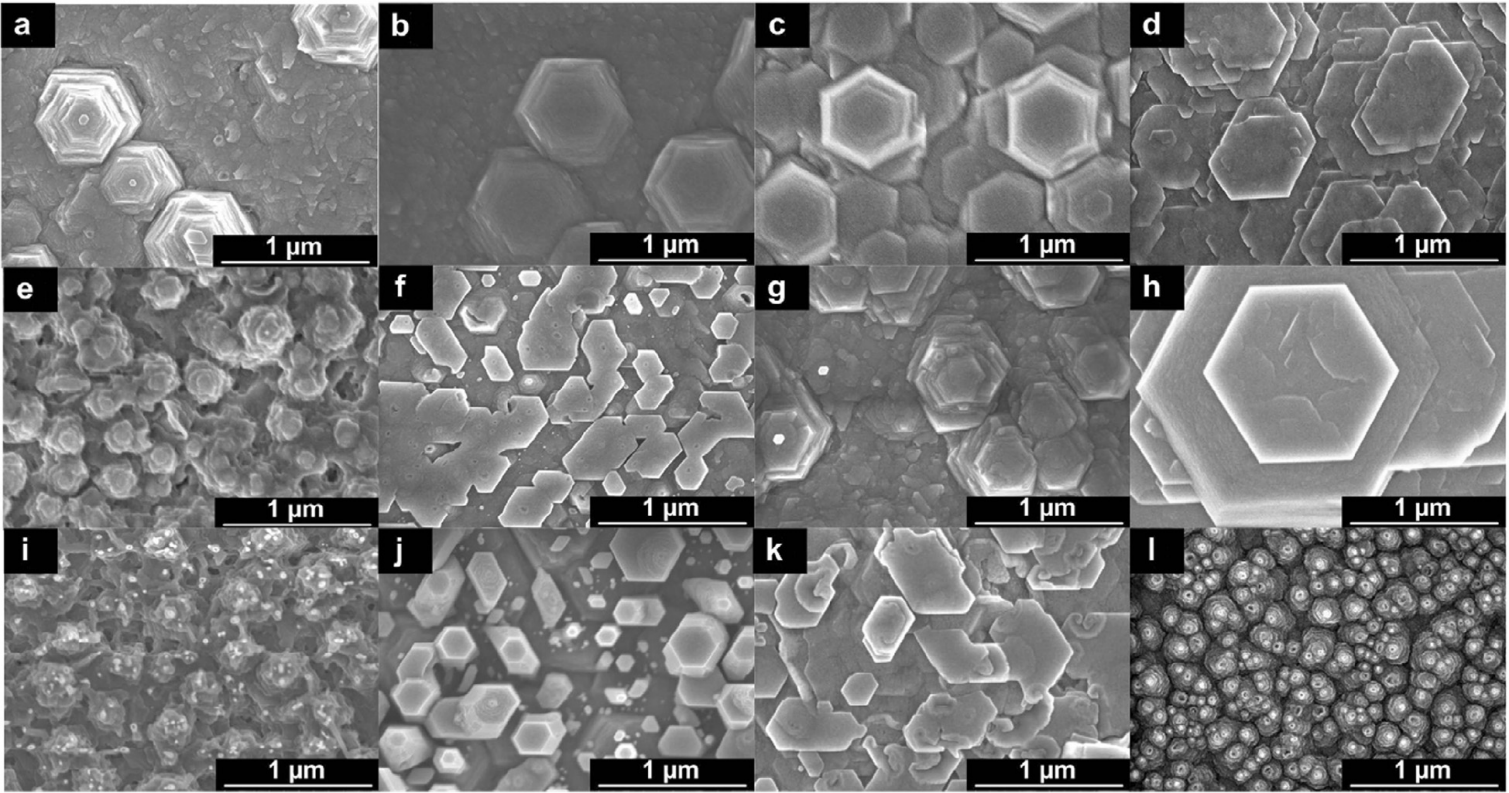
**FESEM images of the top view of ZnO structures after hydrothermal growth.** Images **(a-d)** for the temperature of 70°C with molarities of 2, 20, 30, and 40 mM, respectively; **(e-h)** for the temperature of 80°C with molarities of 2, 20, 30, and 40 mM, respectively; and **(i-l)** for the temperature of 90°C with molarities of 2, 20, 30, and 40 mM, respectively.

**Figure 3:**
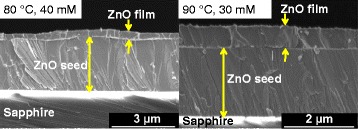
**FESEM images of cross-sectional views of ZnO films after hydrothermal growth.** Images **(a)** for the temperature of 80°C with the molarity of 40 mM and **(b)** for the temperature of 90°C with the molarity of 30 mM.

**Figure 4:**
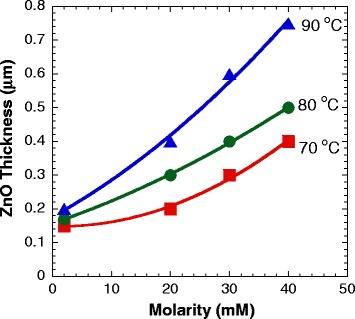
**The dependences of thicknesses of hydrothermally grown ZnO structures on molarities and temperatures.**

Figure 2a,b,c,d,e,f,g,h,i,j,k,l summarizes the FESEM images of top surface views of the samples grown at temperatures of 70°C, 80°C, and 90°C with molarities of 2, 20, 30, and 40 mM. It can be seen that the structures on the top surface with well-defined hexagonal shapes in a few stacking arrangement were grown at low temperatures (70°C to 80°C) and high molarities (30 to 40 mM) as shown in Figure 2c,d,g,h. The best well-defined structure was observed at the temperature of 80°C and the molarity of 40 mM as shown in Figure 2h. It can be assumed that such ranges of temperatures and molarities seem to be the best conditions for the HMTA to play its role as the mineralizer to supply additional hydroxyl (OH^−^) ions in the chemical reaction in defining the shape and morphology of the ZnO structures [14]. However, it can be clearly seen that such a well-defined hexagon-shaped morphological structure was not observed at a high temperature of 90°C for all tested molarities. Figure 3a,b shows the cross-sectional views of samples grown at the temperature of 80°C with the molarity of 40 mM, and at the temperature of 90°C with the molarity of 30 mM, respectively. Although the top surfaces show the morphologies with hill-like structures as shown in Figure 2h,k, it is confirmed from the cross-sectional views that the grown structures are actually thick films with very low heights of such hilly structures on the top surfaces. It is noted here that all hydrothermally grown samples show a thick film structure and their thicknesses increase with temperatures and molarities as illustrated in Figure 4. This simply indicates that the growth rate greatly depends on the temperature and molarity as expected. The interpretation that any change in the structural, optical, and electrical properties of the final structure is contributed by the hydrothermally grown layers seems to be acceptable because the hydrothermally grown layers are thick.

In this study, HMTA and zinc nitrate solution were used as precursors for the growth of ZnO structures. First, HMTA decomposes in water with the assistance of applied heat to generate formaldehyde (CH_2_O) and ammonia (NH_3_), where NH_3_ continues to hydrolyze and produce ammonium (NH_4_^+^) and hydroxyl (OH^−^). Then, OH^−^ reacts with Zn^2+^ to form a complex compound of Zn(OH)_2_. Finally, Zn(OH)_2_ continues to dehydrate into ZnO with the presence of heat. It is worth noting that the supersaturation reaction may also take place. These chemical reactions can be summarized as follows [29-32]:

Decomposition reaction:

$$ {\mathrm{C}}_6{\mathrm{H}}_{12}{\mathrm{N}}_4 + 6{\mathrm{H}}_2\mathrm{O}\to 6\mathrm{C}{\mathrm{H}}_2\mathrm{O} + 4\mathrm{N}{\mathrm{H}}_3 $$

Hydroxyl supply reaction:

$$ \mathrm{N}{\mathrm{H}}_3 + {\mathrm{H}}_2\mathrm{O}\to \mathrm{O}{\mathrm{H}}^{\hbox{-} } + \mathrm{N}{{\mathrm{H}}_4}^{+} $$

Supersaturation reaction:

$$ \mathrm{Z}{\mathrm{n}}^{2+} + 4\mathrm{O}{\mathrm{H}}^{\hbox{-}}\to \mathrm{Z}\mathrm{n}{{\left(\mathrm{O}\mathrm{H}\right)}_4}^{2\hbox{-} } $$

Formation of Zn(OH)_2_:

$$ \mathrm{Z}{\mathrm{n}}^{2+} + 2\mathrm{O}{\mathrm{H}}^{\hbox{-}}\to \mathrm{Z}\mathrm{n}{\left(\mathrm{O}\mathrm{H}\right)}_2 $$

Zn(OH)_2_ dehydration:

$$ \mathrm{Z}\mathrm{n}{\left(\mathrm{O}\mathrm{H}\right)}_2\to \mathrm{Z}\mathrm{n}\mathrm{O} + {\mathrm{H}}_2\mathrm{O} $$

In this work, due to the relatively flat surface of the CVD ZnO seed, the reactions of zinc and hydroxyl ions take place on the entire surface of ZnO seeds which can be also explained by the difference of the surface energies and, hence, lead to the layer-by-layer growth. Due to the positions of the Zn and O ions in the ZnO unit cell and the asymmetry of the hexagonal lattice around the unit cell center, the wurtzite phase of ZnO exhibits a finite dipole moment along the hexagonal *c*-axis. Due to this dipole moment, the ZnO {0001} surfaces become ‘polar surfaces’ and, according to Tasker's rule [33], should be unstable. Nevertheless, the observation of ZnO {0001} surfaces is nothing unusual in the ZnO thin film and nanostructure growth. In general, the stabilization of such polar surfaces can occur via various mechanisms, e.g., surface reconstruction, adsorption of charged atoms/molecules, or internal charge transfer [34-37]. However, the exact mechanisms involved in the stabilization of ZnO {0001} faces are still controversial. Furthermore, caused by this dipole moment, the ZnO {0001} planes have the highest surface energy of all low-index or non-polar planes of the wurtzite ZnO crystal [34]. It is well accepted that surface energies play an important role in the nucleation and growth processes since they have a key influence on the diffusion and adsorption rates of material on a crystal facet.

In general, the deposition and growth of thin films and nanostructures can be explained by the following several consecutive steps that are considered to be applicable not only for vapor-phase regime but also for liquid-phase regime. In the first step, the growth species adsorb on the substrate surface via forming weak bonds due to oscillating (van-der-Waals) or permanent dipole moments. This weak bonding state is referred to as *physisorption* [38]. Due to the weak interaction forces, the potential well, in which the physisorbed molecules are trapped, is shallow and the low-energy barriers at the walls of the well can allow growth species with sufficient energy to escape and ‘hop’ to adjacent sites (wells). The physisorbed molecule at the surface therefore retains some mobility and can move finite distances on the substrate via *surface diffusion* (step two). The mobile molecule can now either desorb from the surface or - and this is the third step - form chemical bonds with the substrate or the growing film/nanostructure, a process referred to as *chemisorption*. In step four, adsorbed growth species accumulate and initiate film/nanostructure growth by *nucleation*. Subsequently, the formed nucleus grows in size and, in the case of thin film deposition, coalesces with nearby nuclei to form a layer. For nanostructure growth, however, the nucleus expands by uni-directional growth which can be imposed by different mechanisms. After the adsorption of growth species on the substrate surface, crystal growth can proceed in different ways depending on the surface energies of the substrate and the growing crystal as well as the interface energy between the two.

**Figure 5:**
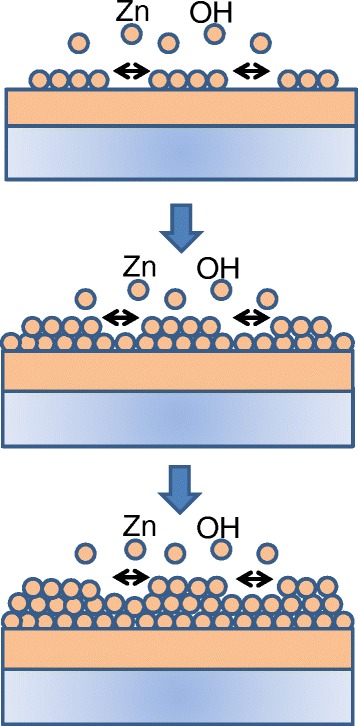
**A schematic of the proposed growth mechanism.**

Since the CVD ZnO seed used in this study is formed in the orientation of ZnO {0001} planes with large grains and considerably flat surfaces, the exposed surfaces are assumed to have the highest surface energies. The differences of surface energies between the polar surfaces and other non-polar surfaces are speculated to be large. Based on the observed morphologies, it seems to justify the assumption that the sum of surface and interface energies of the growing crystal is lower than the seed {0001} surface energy. Correspondingly, minimization of surface energy drives the hydrothermally deposited ZnO to cover the seed surface completely, thus resulting in layer-by-layer growth. Figure 5 illustrates the growth mechanism for the formation of a ZnO continuous film structure. Due to such growth process, a ZnO continuous film structure was obtained and this seems to contribute to the realization of large carrier mobility in the grown film which is described in the next section.

**Figure 6:**
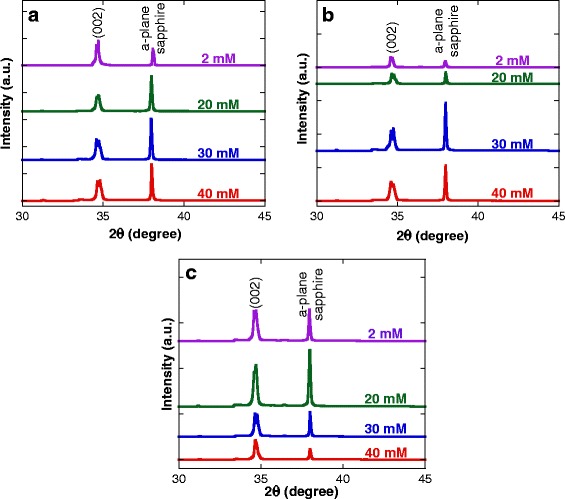
**XRD 2** ***θ*** **scan spectra of ZnO films.** XRD spectra of ZnO films grown at temperatures of **(a)** 70°C, **(b)** 80°C, and **(c)** 90°C, respectively.

Figure 6a,b,c shows the XRD spectra of the samples grown with different molarities at temperatures of 70°C, 80°C, and 90°C, respectively. Two strong peaks in the ranges of 34.51° to 34.71° (ICSD 98-004-0986) and 37.5° to 37.7° (ICSD 98-002-6165) were observed which can be indexed to the ZnO (002) plane and *a*-plane sapphire, respectively. High intensity of the ZnO (002) peak simply indicates that the preferable growth orientation of ZnO structures is along the *c*-axis [39]. It is confirmed that the values of full width at half maximum (FWHM) determined from the ZnO (002) diffraction peak of the hydrothermally grown layers are similar as compared to the corresponding CVD ZnO seeds.

**Figure 7:**
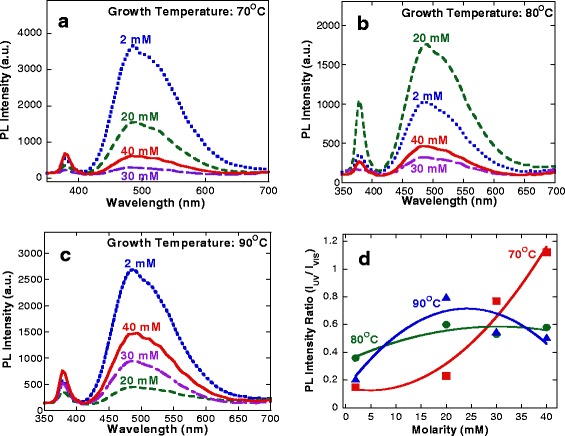
**RT PL spectra of ZnO films.** RT PL spectra of ZnO films grown on CVD ZnO seeds at temperatures of **(a)** 70°C, **(b)** 80°C, and **(c)** 90°C, respectively. **(d)** The ratio of the intensities of both peaks.

Figure 7a,b,c shows the RT PL spectra of the samples grown at growth temperatures of 70°C, 80°C, and 90°C, respectively, with various molarities. Two distinct peaks correspond to NBE emission in the UV region (370 to 380 nm), and a blue-green emission in the visible region (481 to 491 nm) was observed for all samples. The emission in the visible region was reported to be related to the radial recombination of photon-generated holes with single ionized charge of the specific defects such as O vacancies or Zn interstitials [40]. It was reported that the NBE emission could be referred to an intrinsic property of the wurtzite crystal structure of ZnO and originated from the excitonic recombination [41]. Figure 7d summarizes the relative RT PL intensity ratio of the emission in the UV region to the emission in the visible region, *I*_UV_/*I*_VIS_. It can be seen that the ratio linearly increases with the molarities for the sample grown at the temperature of 70°C, but no significant change is observed for the samples grown at the temperatures of 80°C and 90°C. The sample grown at the temperature of 70°C and molarity of 40 mM shows the highest ratio of 1.166. In general, the higher PL intensity ratio means fewer structural defects in the film [42,43]. It is noted here that the original *I*_UV_/*I*_VIS_ values of CVD ZnO seeds are in the range of 0.35 to 0.65. From these results, it can be said that the grown hydrothermal ZnO layers seem to have similar or better quality than CVD ZnO seed layers based on the comparison of *I*_UV_/*I*_VIS_ values.

**Figure 8:**
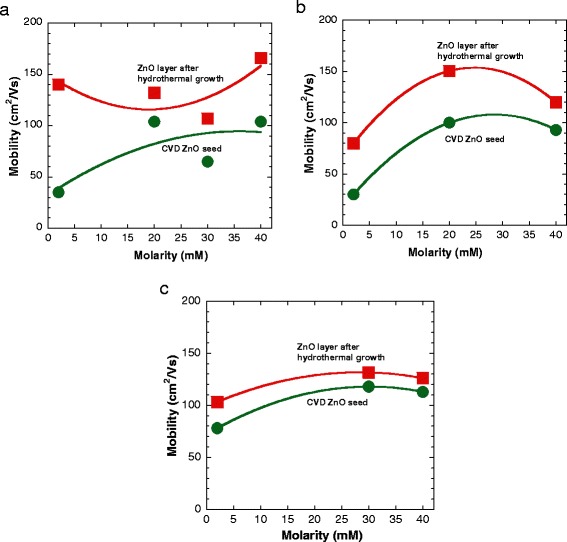
**Measured electron mobility of the ZnO layer after hydrothermal growth.** Electron mobility of the ZnO layer at temperatures of **(a)** 70°C, **(b)** 80°C, and **(c)** 90°C. Data of the CVD ZnO seed was also included for comparison.

**Figure 9:**
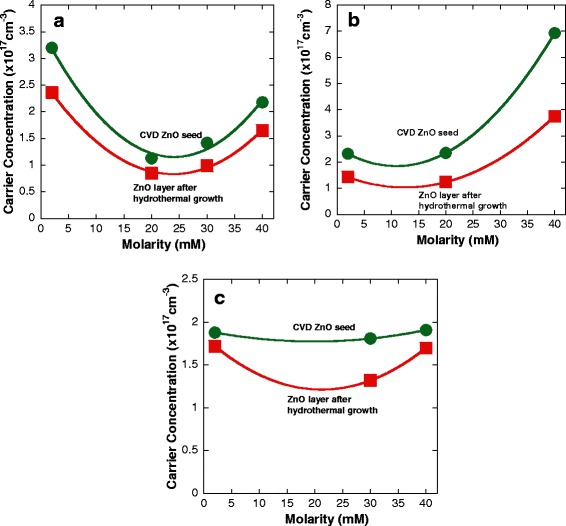
**Measured carrier concentration of the ZnO layer after hydrothermal growth.** Carrier concentration of the ZnO layer at temperatures of **(a)** 70°C, **(b)** 80°C, and **(c)** 90°C. Data of the CVD ZnO seed was also included for comparison.

The Hall effect measurements in a Van der Pauw configuration were performed to study the electrical properties of the grown samples. As shown in Figure 8a,b,c, the electron mobilities after the hydrothermal growth show a large increase as compared to the corresponding mobilities of CVD ZnO seeds. The highest mobility of 166 cm^2^/Vs was obtained at the molarity of 40 mM and temperature of 70°C with the increment percentage of 60%. Figure 9a,b,c summarizes the corresponding carrier concentration of CVD ZnO seeds and ZnO layers after hydrothermal growth. The carrier concentrations were confirmed to show the decrement in value for the hydrothermally grown ZnO layers as compared to the corresponding concentration of CVD ZnO seeds. These results suggest that a ZnO layer with a low carrier concentration can be realized by a hydrothermal deposition, resulting in an increase of carrier mobility. The corresponding carrier concentration for the sample with the highest mobility is 1.65 × 10^17^ cm^−3^. In general, a low carrier concentration is suitable for further *p*-type and LED engineering [12].

## Conclusions

In conclusion, the hydrothermally grown ZnO in the form of a thick film structure was obtained instead of nanostructures due to the flat and large grain surface structure of seed layers. Such a surface structure of the seed layer has promoted the lateral growth of ZnO instead of vertical growth. The grown ZnO layers were confirmed to be hexagonal wurtzite structures and highly oriented towards the *c*-axis. The increases of mobility were obtained for all grown ZnO layers as compared to the corresponding mobilities of CVD ZnO seeds. The highest mobility of 166 cm^2^/Vs at the considerably low carrier concentration of 1.65 × 10^17^ cm^−3^ was achieved.
